# The Intestinal Microbiota: Impacts of Antibiotics Therapy, Colonization Resistance, and Diseases

**DOI:** 10.3390/ijms22126597

**Published:** 2021-06-20

**Authors:** Taif Shah, Zulqarnain Baloch, Zahir Shah, Xiuming Cui, Xueshan Xia

**Affiliations:** 1 Faculty of Life Science and Technology, Kunming University of Science and Technology, Kunming 650500, China; taifshah@yahoo.com; 2 Yunnan Key Laboratory of Sustainable Utilization of Panax Notoginseng, Kunming 650500, China; 3 Faculty of Animal Husbandry and Veterinary Sciences, College of Veterinary Sciences, The University of Agriculture Peshawar, Peshawar 25120, Pakistan; drzahir@aup.edu.pk

**Keywords:** intestinal microbiota, antibiotics, pathogens, colonization resistance, diseases

## Abstract

Trillions of microbes exist in the human body, particularly the gastrointestinal tract, coevolved with the host in a mutually beneficial relationship. The main role of the intestinal microbiome is the fermentation of non-digestible substrates and increased growth of beneficial microbes that produce key antimicrobial metabolites such as short-chain fatty acids, etc., to inhibit the growth of pathogenic microbes besides other functions. Intestinal microbiota can prevent pathogen colonization through the mechanism of colonization resistance. A wide range of resistomes are present in both beneficial and pathogenic microbes. Giving antibiotic exposure to the intestinal microbiome (both beneficial and hostile) can trigger a resistome response, affecting colonization resistance. The following review provides a mechanistic overview of the intestinal microbiome and the impacts of antibiotic therapy on pathogen colonization and diseases. Further, we also discuss the epidemiology of immunocompromised patients who are at high risk for nosocomial infections, colonization and decolonization of multi-drug resistant organisms in the intestine, and the direct and indirect mechanisms that govern colonization resistance to the pathogens.

## 1. Overview of the Microbiota

Humans and other mammalian gastrointestinal tracts (GIT) are home to trillions of microorganisms, such as bacteria, archaea, fungi, protozoa, helminths, and viruses, collectively named the microbiota [1]. Consequently, there is great diversity in the microbial composition between and within microbiota members. Despite its diversity, most intestinal microbiota are comprised of four phyla, i.e., *Actinobacteria*, *Bacteroidetes*, *Firmicutes*, and *Proteobacteria*. The *Bacteroidetes* and *Firmicutes* phyla account for the greatest population (more than 90%) in the colon. We will briefly discuss each of these phyla below and highlight important members to understand the intestinal microbiota composition [2].

Phylum *Actinobacteria* comprises aerobic, anaerobic, Gram-positive bacteria with high G + C content in their genomic DNA. The long-term coexistence of certain bacterial species, such as *Bifidobacteria* spp. (also known as probiotics), which adhere to the intestinal mucosa, results in a mutually beneficial relationship. The World Health Organization has defined probiotics as live microorganisms that provide a health benefit to the host when administered in sufficient amounts [3]. Probiotics protect the host from pathogens through various activities, including competitive exclusion, bile salt hydrolase activity, nutrient metabolism assistance, and immune and digestive system regulation. On the other hand, the host provides nutrient-rich niches to ensure microbiota survival [4].

Phylum *Bacteroidetes* comprises aerobic, anaerobic, non-spore-forming, Gram-negative, rod-shaped bacteria that colonize the intestine. *Bacteroides* is one of the most predominant genera colonizing the intestinal tract [5]. Members of this genus are known to digest complex carbohydrates that are resistant to the host’s digestive enzymes. After the breakdown of complex polysaccharides, the host uses small fatty acids like acetate, propionate, and butyrate for energy [6].

Phylum *Firmicutes* comprises obligate facultative anaerobic, Gram-positive endospore-forming bacteria. Endospores are dormant, non-reproductive structures that allow bacteria to survive under adverse conditions, such as nutrient deficiency, ultraviolet radiation, desiccation, extreme temperature, and chemical disinfectants, for a long time and become active when the environment becomes favorable. *Clostridia* within this phylum contain diverse bacteria that live in the intestine and play several beneficial roles. Members of this bacterial class colonize between mucosal folds to establish close relationships with intestinal epithelial cells and produce butyrate as an end product of fermentation. Members of this class also promote host immune homeostasis in the intestine by activating colonic immune cells. The *Clostridia* class also includes important bacterial pathogens (*Clostridium perfringens*, *Clostridium tetani*, and *Clostridium difficile*) that cause various human diseases. *Bacilli* members (*Enterococcus* and *Streptococcus* spp.) are clinically important, oxygen-tolerant, generally found in low abundance, undergoing pathogenic expansion during intestinal dysbiosis.

Phylum *Proteobacteria* comprises Gram-negative bacteria that are both obligate and facultative. *Lactobacillus* spp. and *Escherichia coli* Nissle adhered to the intestinal mucosa and showed a mutually beneficial relationship. According to some studies, the increased prevalence of *Proteobacteria* in the microbial community can be diagnosed as dysbiosis and diseases [7]. Pathogenic *E. coli* strains and *Klebsiella* are found in low abundance in the *Enterobacteriaceae* family of *Proteobacteria*. In addition to probiotics, antibiotics have been shown to improve enteric pathogen colonization in the intestine. For example, changes in the composition of the intestinal microbiome can increase susceptibility to *Salmonella enterica* subsp. *Typhimurium* and *C. difficile* infections [6].

## 2. Impact of Antibiotic Therapy on Microbe Colonization and Diseases

**Figure 1:**
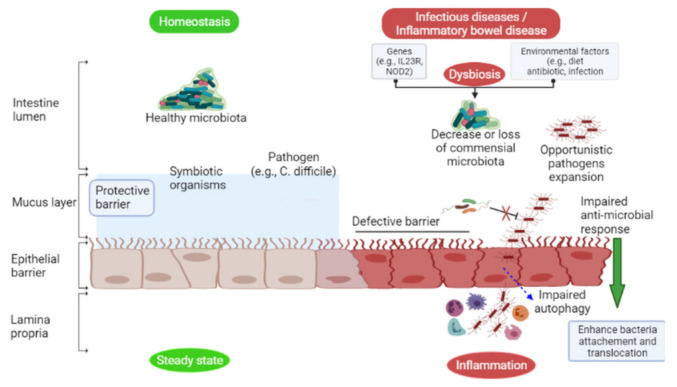
Under homeostatic conditions (**left** green), certain Gram-negative commensal bacteria induce IgA and IgG antibody production from B cells, recognizing Gram-negative bacteria surface antigens (flagellin, LPS) in the intestinal lumen, thus contributing to host defense against symbionts and pathogens. Symbiotic organisms stimulate mucus production to prevent enteric pathogens from colonizing the intestinal mucosa. However, during dysbiosis (**right** red), a decrease or loss of commensal bacteria in the intestine may result in opportunistic pathogens (e.g., *C. difficile*) infection due to the secretion of virulence factors (toxins) that damage and breach the epithelial layer, causing inflammation.

Intestinal microbes are stable under physiological conditions, but antibiotics administration, nutrients availability, physical stress, and host factors can all cause dysbiosis in the microbiota (Figure 1). Dysbiosis is characterized by a reduction in the diversity of microbes and the normal function of the intestinal microbiota in maintaining host wellness. In addition, it may cause loss of specific microbial populations that dysregulate the production of antimicrobial peptides or metabolites against pathogen colonization [8,9].

Antibiotics are used for the treatment of potentially life-threatening bacterial diseases. Previous studies have shown that incorrect or prolonged antibiotics use may lead to the elevation of unanticipated and undesirable microbial communities in the intestines. Theoretically, an antibiotic can only affect the intestinal microbiota composition through direct exposure, which is only possible if the antibiotic reaches the intestinal lumen. Therefore, antibiotics taken orally are delivered directly to the intestinal lumen. Antibiotics circulate in the bloodstream and eventually reach the liver, where they may be modified; depending on the nature of the antibiotic, it may be excreted into the bile or returned to the bloodstream as waste products for renal clearance. Several factors influence antibiotic absorption in the intestines, including antibiotic properties, intestinal membrane integrity, and transport mechanisms. Antibiotics are absorbed in the intestinal lumen, reducing exposure to the microbiota. For example, orally administered metronidazole was entirely absorbed in the small intestine [6], resulting in significantly lower intestinal metronidazole concentrations as it passes through the GIT without disturbing the intestinal microbiota. In contrast, poorly absorbed antibiotics in the intestine, such as vancomycin, remain at high concentrations in the GIT after oral administration, implying that oral metronidazole had a lower impact on the microbiota than oral vancomycin. Thus, antibiotic activity and intestinal absorption determine how antibiotics affect the composition of the microbiota and host susceptibility to pathogens. These key factors, among others, can be considered when selecting antibiotics and determining their effects on the intestinal microbiota.

### 2.1. Clinical Consequences of Antibiotic Treatment

Antibiotics may alter the composition of the microbiota in the intestines. The restoration of microbial diversity in children after antibiotic treatment has been reported to take about one month [10]. Gentamicin, meropenem, and vancomycin administration increased the prevalence of *Enterobacteriaceae* and other pathobionts in adults while decreasing the *Bifidobacterium* species [11]. Although the intestinal microbiota’s baseline composition was restored within 45 days, and several bacteria were undetectable for the remaining 180 days [11]. Antibiotics can also disrupt the balance of the bacterial species in the intestines. For example, reduced bacterial diversity promotes the growth and invasion of enteric pathogens, especially *C. difficile* [12]. Several previous studies assessing the effect of antibiotic therapy on intestinal microbiota are listed in Table 1.

#### 2.1.1. Antibiotic-Associated Diarrhea

Some antibiotics thin the mucus layer and disrupt tight junctions, exposing the intestinal epithelium to damage. Antibiotic-associated diarrhea affects approximately 5–35% of patients following antibiotic treatment [13]. Antibiotic-associated diarrhea occurred in 17.5% of adult patients after receiving antibiotics for 5–10 days [14]. Antibiotic-associated diarrhea caused by *C. difficile* is more severe than antibiotic-associated diarrhea caused by non-*C. difficile* [15]. Probiotics therapy (*Lactobacillus rhamnosus* and *Saccharomyces boulardii* is recommended in the 2016 European Society for Pediatric Gastroenterology, Hepatology, and Nutrition guidelines to prevent antibiotic-associated diarrhea [16].

#### 2.1.2. *C. difficile*-Associated Diarrhea

*C. difficile* is an obligate anaerobe, spore-forming bacteria that commonly infects immunocompromised peoples, such as hematopoietic stem cell transplant recipients [17]. These bacterial spores are typically consumed orally and proliferate in the colon of susceptible hosts. Under favorable conditions, these bacteria produce toxins in the host that disrupt the intestinal epithelial barrier and cause diarrhea [18,19]. Antibiotics are the most important risk factor for *C. difficile* nosocomial infection, with the most commonly associated drugs are ampicillin, amoxicillin, cephalosporin, clindamycin, and fluoroquinolone. The duration and number of antibiotics taken increase the risk of *C. difficile* infection. Other risk factors include age, weak body immunity, and being hospitalized [20]. Metronidazole 500 mg three times a day for ten days is recommended in mild and moderate cases and vancomycin 125 mg four times a day for ten days in severe cases. Antibiotics that caused *C. difficile* infection should be discontinued [21].

#### 2.1.3. Helicobacter Pylori Infection

*Helicobacter pylori* (*H. pylori*) is a Gram-negative bacterium that colonizes the human GIT and causes inflammation. In most cases, the inflammatory response is mild and asymptomatic; however, the infection can occasionally cause intestinal metaplasia, gastric cancer, and gastric and duodenal ulcers [15]. Quadruple bismuth is recommended as first-line therapy in areas with high clarithromycin and metronidazole resistance [22]. *H. pylori* eradication can have both positive and negative effects on the host. It restores microbiota composition [23] and causes changes in the microbiota composition that affect the host [24]. *H. pylori* eradication was associated with a decrease in the relative abundance of *Bacteroidetes* and an increase in *Firmicutes*. The addition of probiotics (*S. boulardii*) to triple therapy was more effective for *H. pylori* eradication [25]. Probiotics (*Lactobacillus* and *Bifidobacterium*) combined with *H. pylori* eradication therapy were more effective and safer than therapy without probiotics [26].

#### 2.1.4. Antibiotic Therapy Cause Obesity, Asthma, Allergy, and IBD

The use of antibiotics in infants and children has been linked to several long-term clinical consequences, including obesity, asthma, and allergies [15]. Antibiotic exposure during childhood (particularly between the ages of 6 and 12 months) delayed intestinal microbiota development [27] and decreased *Enterobacteriaceae*, *Lachnospiraceae*, and *Erysipelotrichaceae* species in the intestines [27]. In addition, early antibiotic exposure causes dysbiosis, which aids in the pathogenesis of inflammatory bowel disease (IBD). According to a population-based cohort study, infants who received antibiotics in their first year of life were more likely to be diagnosed with IBD than untreated individuals [28].

### 2.2. Multi-Drug-Resistant (MDR) Organisms Are Found in the Intestinal Microbiota

Resistomes are microbiota in the intestines that carry multiple antibiotic-resistance genes [29]. There are two types of resistomes in the intestinal microbiota: the resident resistome (commensal bacteria carrying antibiotic-resistance genes) and the transitory resistome (antibiotic-resistance genes carried by bacteria periodically). The transitory resistome can either transfer its resistance gene to commensal bacteria or become a permanent microbiota member [30]. Therefore, characterizing the intestinal microbiota in humans that carry the antibiotic resistance gene and understanding how the antibiotic resistance genes can transfer among different commensal members and opportunistic pathogens is of great interest [31].

The high prevalence of *C. difficile* opportunistic infection may be due to the emergence of antibiotic-resistant strains [32,33,34]. Antibiotic-induced perturbations of the intestinal microbiota result in the loss of colonization resistance in hosts, making them vulnerable to *C. difficile* infection [35]. This concern mainly arises in hospitals, where *C. difficile* can quickly spread from an infected person to a healthy person, making it a leading cause of nosocomial infection in developed countries [18,19]. In addition to *C. difficile* infection, *Klebsiella pneumoniae* is an opportunistic pathogen that serves as an infection reservoir. *K. pneumoniae* is a member of the healthy human intestinal microbiota and a leading cause of hospital-acquired infections. However, the mechanisms that promote colonization to infection are poorly understood. *K. pneumoniae* GIT carriage was a risk factor for infection in hospitalized patients [36]. In a study, Stercz and his colleagues investigated the effect of antibiotics (ampicillin, ciprofloxacin, ceftazidime) on the intestinal colonization of CTX-M-15 ESBL and OXA-162 carbapenemase-producing *K. pneumoniae* using the mice model [37]. *K. pneumoniae* colonization increased after ampicillin and ceftazidime treatments. In contrast, ciprofloxacin treatments decreased colonization. The gene *blaOXA-162* was correlated with *K. pneumoniae* in vivo, whereas the copy numbers of the gene *blaCTX-M-15* increased from the first to the fifteenth day after ceftazidime treatment. These findings show that antibiotics have various effects on bacterial colonization, antibiotic resistance genes, and the persistence of *K. pneumonia*. Furthermore, colonization of MDR bacteria in the intestines has been identified as a risk factor for severe disease in patients undergoing hematopoietic stem cell and liver transplantation [38,39].

## 3. Nosocomial Infections of the GIT

The intestinal microbiota contains a large number of opportunistic pathogens. The microbiota’s role in protecting immunocompromised patients from opportunistic pathogens is critical in the hospital. These opportunistic pathogens can cross the intestinal barrier (Figure 1) and cause infections after fecal contamination of skin, intravenous lines, or other body sites [40]. Hospitalized patients, such as those receiving chemotherapy or undergoing hematopoietic stem cell transplantation, are at higher risk of *C. difficile* infection [41]. Antibiotic treatment can prevent these infections; however, antibiotic administration can disrupt the intestinal microbiota and develop antibiotic-resistant bacteria strains [42]. *C. difficile* colonization causes gastrointestinal infection [43], and other nosocomial pathogenic bacteria can translocate and enter the bloodstream to cause systemic infections [44].

## 4. Bloodstream Infections Originate from the GIT Colonization

Bloodstream infection in immunocompromised patients originates from the GIT due to changes in the microbiota or damage to the mucosal barrier, mediated by many factors, e.g., exposure to chemotherapy, radiation, or antibiotics that systemically disseminate intestinal bacteria [45]. The most common bacteria translocating are oxygen-tolerant pathobionts such as vancomycin-resistant *Enterococcus* (VRE), *E. coli*, *Klebsiella*, and *viridans streptococci* [46]. Chemotherapy, which is used to treat cancer, suppresses the immune system. It includes various drugs that inhibit various cellular mitosis steps, affecting rapidly dividing cells (cancer cells). Specialized stem cells in the GIT [47] and hematopoietic stem cells in the bone marrow [48] divide rapidly under healthy homeostatic conditions. These cells become vulnerable to chemotherapy antimitotic effects, which disrupt the normal turnover of these cells. The specialized stem cells in the GIT normally replenish the mucosal epithelial cells to maintain their integrity. On the other hand, chemotherapy prevents the replacement of aging and damaged intestinal epithelial cells lining. Damaged cells produce reactive oxygen species, which initiates a repair response that activates transcription factors in the mucosal epithelial cells. Chemotherapy-induced inflammatory damage cell apoptosis in the GIT mucosa often results in painful lesions and a compromised mucosal barrier [49]. Chemotherapy also affects hematopoietic stem cells in the bone marrow, which give rise to immune blood cells.

Neutrophils are the most abundant among the white blood cells with a short life span that act as a primary defense response to infections [50]. Promyelocytes are the earliest recognizable precursors of neutrophils, which actively synthesize DNA and are susceptible to the antimitotic effects of chemotherapy. Their offspring develop into myelocytes, the numerous proliferating neutrophil precursors, and thus the most severely affected cells by chemotherapy. Chemotherapy-induced neutropenia (low neutrophil count) can predispose a patient to infections. Broad-spectrum antibiotics are commonly used in immunocompromised patients to prevent opportunistic infections. As a result of antibiotic therapy, immunocompromised patients are more susceptible to bacterial infections from the GIT. Infected persons may develop neutropenia, which impairs their immune response to pathogens. Antibiotics treatment in combination with chemotherapy can promote intestinal dysbiosis and antibiotic-resistant bacterial strains. The patients may also develop mucositis, which allows the intestinal bacteria to enter the bloodstream and cause systemic infection.

## 5. Intestinal Microbiota Modulation by Fecal Microbiota Transplantation (FMT) for the Decolonization of MDR Organisms

MDR organisms in the intestines of hospitalized patients can be opportunistic pathogens due to antibiotic pressure. Patients colonized with MDR organisms from the GIT or transmitted from other individuals are at a higher risk of infection. These pathogens can enter the body through the damaged intestinal barrier and cause local or systemic infections. Intestinal microbiota plays a vital role in preventing these infections, which have gained attention in using FMT as a preventive strategy to reduce MDR organism carriage. FMT is strongly recommended for patients with recurrent *C. difficile* infection [51] and who have not responded to appropriate antibiotic treatment [52]. Oral capsules containing lyophilized fecal microbiota administered through colonoscopy were equally effective as frozen products [53]. FMT is highly effective [52], although cases of bacteremia following FMT administration have been reported [54]. A rise in the prevalence of *C. difficile* infection has been observed in pediatric patients [55], particularly in children who received prolonged antibiotic therapy or those with chronic IBD, oncological conditions, or who have recently undergone surgery [56]. According to current evidence, the recipient’s age does not affect FMT efficacy or safety. Disruption of the intestinal microbiota following prolonged antimicrobial therapy contributes to the pathogenesis of graft-versus-host disease and overall mortality in hematopoietic stem cell transplantation recipients [57,58]. Based on these findings, FMT may help to prevent hematopoietic stem cell transplant-related mortality. FMT has been proposed to eradicate drug-resistant bacteria from the intestinal microbiota. Adult patients who received prolonged antibiotic therapy for chronic *C. difficile* infection showed a higher proportion of antibiotic resistance genes in their intestinal microbiota than healthy adults; however, the number and diversity of antibiotic resistance genes decreased after FMT administration [59,60]. Antimicrobial-resistant genes can also be acquired from FMT donor stool, highlighting the importance of healthy stool donor selection and the need for further standardization [61]. The carbapenem-resistant *Enterobacteriaceae* and VRE were the most frequently isolated pre-FMT MDR bacteria from MDR-infected patients [62] and immunocompromised patients [63]. Other MDR organisms identified are *Pseudomonas aeruginosa*, methicillin-resistant *Staphylococcus aureus*, and *Acinetobacter*. FMT therapy is commonly given through the nasoduodenal tube, nasogastric tube, oral capsules, or colonoscopy. The donor’s age is specified as being between 6 and 60 years. Patients undergoing FMT must fast for at least 12 h, begin treatment with a proton pump inhibitor twice a day to neutralize gastric acid, and discontinue taking antibiotics. FMT was associated with severe adverse reactions in a hospitalized patient with known liver cirrhosis and recurrent hepatic encephalopathy [64]. Other studies reported mild diarrhea, mild abdominal discomforts, food intolerance, constipation events after FMT administration.

## 6. The Intestinal Microbiota Showed Colonization Resistance to Pathogens

The concept of colonization resistance refers to the ability of commensal microbiota to prevent the colonization and overgrowth of pathogenic bacteria [65]. Colonization resistance is an intestinal defense mechanism that occurs due to direct competition between commensals and pathogens for the intestinal niche and nutrition. The intestinal microbiota plays a critical role in excluding invading bacteria and inhibiting the growth of enteric pathogens. Several studies elucidated the molecular mechanisms of excluding invading pathogens and inhibiting the growth of enteric bacteria within the GIT using different pathogens and antibiotic regimens. Key members of the bacterial species contributing to colonization resistance against pathogens are *C. difficile*, VRE, *L. monocytogenes*, *E. coli*, and *Enterococcus faecalis* [66,67,68,69].

Studies have also shown that certain commensal microbiota inhibits the growth of MDR bacteria, although the mechanism by which microbiota influence pathogen colonization is unknown. VRE (obligate anaerobe *Barnesiella*) did not colonize intestines in mice [70,71]. Mice administered with a specific bacteria consortium (*Blautia*, *Clostridium bolteae* prevented VRE colonization and cleared persistent VRE [67]. Four bacterial species (*Coprococcus*, *Desulfovibrio*, *Oscillospira*, and *Parabacteroides* species) were depleted from the microbiota of adult patients colonized by extended-spectrum β-lactamase (ESBL)-producing bacteria [72]. In contrast, these four bacterial species were present in the microbiomes of patients who were not colonized by ESBL-producing bacteria. Hence, it is clear that beneficial bacteria provide colonization resistance to pathogens by two mechanisms: direct competition between commensals and pathogens for nutrients uptake or physical environment (niches) establishment and indirect mechanisms of colonization resistance, derived from the commensal bacteria stimulated immune system. In this section, we will discuss mechanisms by which intestinal microbiota contribute to pathogens’ colonization resistance.

### 6.1. Direct Mechanisms of Colonization Resistance

**Figure 2:**
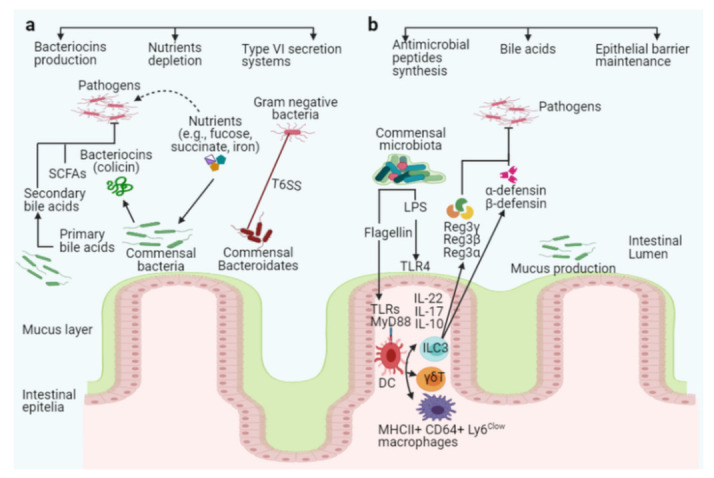
The intestinal microbiota acts as a barrier against enteric pathogens via direct and indirect colonization resistance mechanisms. Direct colonization resistance mechanisms (**a**): Several commensal *Bacteroidetes* prevent pathogens from colonizing the intestinal mucosa, and the commensal *E. coli* Nissle strain consumes nutrients, limiting nutrients availability to specific pathogens (pink color with flagella). The probiotic *E. coli* Nissle strain can also absorb iron, limiting its availability to the pathogen *S. Typhimurium*. Commensal bacteria secrete antimicrobial peptides, such as bacteriocins (colicin), SCFAs, and T6SS, to target and kill invading pathogens. Together with antimicrobial peptides, commensal bacteria produce enzymes that convert conjugated primary bile acids to secondary bile acids (toxic to invading pathogens). Indirect colonization resistance mechanisms (**b**): Surface antigens such as flagellin or LPS from commensals bacteria stimulate host innate immunity via TLRs and MyD88 on epithelial or dendritic cells (DCs). ILC3, γδT, and Th17 cells can be activated to produce interleukin IL-10, IL-17, and IL-22, which promotes secretion of the antimicrobial peptides Reg3γ, Reg3β, Reg3α, α-defensin, and β-defensin from epithelial cells to inhibit pathogen colonization in the intestines (pink color with flagella).

The microbiota promotes direct colonization resistance to pathogens through killing and competition for resources. One mechanism is directly competing for the same niche where intestinal bacteria provide colonization resistance to pathogens (Figure 2a). Some microbiota acquires nutrients more efficiently than pathogens, impeding pathogen replication and colonization in the intestines, while others secrete antimicrobial peptides that directly kill pathogens. These targeted killing mechanisms compete for nutrients and niches between closely related bacterial spp. The following sections discuss the aspects of direct mechanisms of colonization resistance between beneficial and harmful bacteria.

#### 6.1.1. Killing or Suppression of Pathogens Through Antimicrobial Peptides

Killing or growth suppression can play a significant role in pathogens’ colonization resistance [73]. Bacteriocins are small peptides produced by bacteria in their environment that have antimicrobial activity. Gratia discovered the first bacteriocin, colicin, in 1925 [74]. Since then, many bacteriocins have been isolated and characterized from different bacterial species, including human and animal intestinal bacteria, lactic acid bacteria of fermented foods, and *Bifidobacteria* [75]. The molecular weight of microcin is less than 10 kDa, whereas the molecular weight of colicin is greater than 20 kDa. Compared to non-producer control strains, bacteriocin (colicin) producing *E. coli* strains demonstrated improved and prolonged persistence in mice [76]. However, streptomycin pre-treatment resulted in a disrupted *E. coli* and bacteriocin-producing *E. faecalis* community in these mice. These bacteria were more capable of colonizing antibiotic-treated mice than mice that had not been pre-treated with antibiotics. Bacteriocin-producing bacteria may also inhibit the colonization of opportunistic pathogens such as *E. faecalis* and VRE [69].

Microcins are bacteriocins produced by Gram-negative bacteria that have limited antimicrobial activity against other Gram-negative bacteria. Human probiotic *E. coli* Nissle 1917 produced microcins in mice to protect them from *S. Typhimurium* infection [77]. In a study, mice administered water supplemented with different bacteriocin-producing bacterial strains inhibited the growth of pathogenic *Clostridium* and *Staphylococcus* strains without affecting the beneficial microbiome [78]. Hence, it is clear that bacteriocins from the *Enterobacteriaceae* play a significant role in colonization resistance to bacterial pathogens without causing significant disruptions in the intestinal microbiota population.

#### 6.1.2. Metabolites from Intestinal Microbiota Inhibit Pathogenic Bacteria

Metabolites produced by intestinal microbiota inhibit the growth of pathogenic bacteria. Short-chain fatty acids (SCFAs) produced by healthy intestinal microbiota are protective against enteric pathogens. Intestinal microbiota ferment unabsorbed starch, soluble dietary lipids, and vitamins. Intestinal microbiota members *Bacteroidetes* and *Clostridia* produce SCFAs in adults [73]. *Bacteroidetes* are the primary fermenters, transforming sugars from complex carbohydrates to organic acids and SCFA, while *Clostridia* uses the organic acids to generate additional SCFAs. SCFAs have direct antimicrobial activity by diffusing through bacterial membranes and lowering intracellular pH. SCFAs have promising applications in human health and food safety due to their antimicrobial activity [79]. *Clostridia* SCFAs are important metabolites that inhibit *S. Typhimurium* growth in mice intestines [73], pathogenic *E. coli* strain growth in streptomycin-treated mice [80], and *C. difficile* growth in humans [81,82]. The intestinal microbiota secretes bile acids, which are amphipathic, cholesterol-derived molecules. Bile acids are synthesized in the liver and then conjugated with taurine or glycine before being stored in the gall bladder. Subsequently, the bile acids are secreted into the small intestine, where they emulsify fat and fat-soluble vitamins for absorption. Nearly 95% of bile acids are reabsorbed at the distal ileum in humans, and the remaining 5% converted or deconjugated to secondary bile acids, such as deoxycholic acid and lithocholic acid [83]. *Clostridium perfringens* and *Clostridium scindens* secrete bile salt that is reabsorbed in the intestines and transported back to the liver for conjugation [84]. Secondary bile acid that the host has not absorbed is excreted [85]. It has been proposed that bile salt-producing bacterial species (*Lactobacilli*, *Bifidobacteria*, *Clostridia*, *Bacteroides*) that are resistant to bile acid toxicity have a better chance of survival [86]. Deconjugation by commensals *Lactobacillus* and *Clostridium* ensures colonization resistance to pathogenic bacteria [66]. The secretion of deoxycholic acid from *B. bifidum* reduced the pathogenicity of *enterobacteria* and *Vibrio cholerae* by targeting the virulence-associated effectors T6SS and T3SS [87]. Pathogens spread in the absence of deoxycholic acid-producing commensals, as seen in the case of *C. difficile* enterocolitis [88]. Increased bile acid levels (through therapy or diets) promote the growth of *Firmicutes* and *Clostridium* species involved in bile acid deconjugation and inhibit *Bacteroidetes* and *Actinobacteria*. Specific bacterial pathogens, such as *S. Typhimurium*, can survive in the presence of high bile acid concentrations for extended periods [87]. Excess bile acids, such as deoxycholic acid, can lead to cholesterol gallstones and colon cancer, and the intestinal microflora plays an additional role in these complications. Secondary bile acids have antimicrobial properties, as they alter the integrity of the microbial cell membrane, causing spillage of intracellular contents and thus inhibiting the growth of bile acid-intolerant microbes [89,90]. These antimicrobial agents promote the growth of the intestinal microbiota while also protecting the host from various pathogens.

#### 6.1.3. Competition for Shared Niches and Nutrients

Competition between commensals and pathogens for niches and nutrients provides colonization resistance to pathogens in the intestines [9]. The intestinal epithelium monolayer cells are tightly connected, which plays a complex role in mucus production and immune response [91]. Commensals attach to the intestinal mucus from birth, occupying all available space to prevent enteric pathogens colonization [92]. The mucin-rich mucus layer contains O-glycan peptide motifs that act as receptors for commensals like *Bifidobacteria* and *Lactobacilli* colonization [93]. Mucin inhibits pathogenic bacterial attachment, biofilm formation, virulence factor detoxification, and commensal survival during competition with pathogens [94]. As a result, mucus protects the natural intestinal environment and acts as the first line of defense against bacterial invasion. Commensals like *E. coli* and *Bifidobacteria* secrete mucins to protect themselves from pathogenic *E. coli* strain and *C. difficile* invasion [95,96]. Some enteric pathogens, such as *S. Typhimurium*, *C. difficile*, pathogenic *E. coli* strain O157:H7, *H. pylori*, *Campylobacter*, and *V. cholerae,* have evolved specific mechanisms, including flagella motility or lack of adhesins required for bacterial adhesion to avoid mucus [95]. Other virulence mechanisms used to kill commensal competitors include type III or VI secretion systems (T3SS, T6SS) from *V. cholerae* [97,98], mucin-binding proteins from *Listeria monocytogenes* [99], and fimbriae adhesins from *S. Typhimurium* [100]. Hence, it is clear that the mucus layer is essential in preventing pathogenic bacteria and could be a future target for anti-diarrheal drugs. Competition for available nutrients is a mechanism by which commensals provide colonization resistance to pathogens in the human intestines [9]. The use of six sugar molecules by human commensal *E. coli* HS and seven molecules by probiotic *E. coli* Nissle limit nutrient availability to pathogenic *E. coli* O157:H7, thereby inhibiting their colonization in mice [101]. Sometimes pathogenic *E. coli* O157:H7 can evade this mechanism and start sugar utilization that commensal *E. coli* failed to utilize [102]. Pathogens use the nutrition provided by commensal bacteria, and those pathogens unable to utilize these resources are eliminated. For example, pathogenic *C. difficile* survives by metabolizing succinate from the symbiont *Bacteroides thetaiotaomicron*, whereas *C. difficile* mutant strains that cannot use this source were eliminated ([103]. The fucosylation of glycan induced by *Bacteroides* provides a nutrition source to symbionts for their successful colonization. This mechanism is vital for the colonization of *Bacteroides* and pathogenic bacteria, both of which require fucosis to survive [104,105,106]. Pathogenic bacteria (*S. Typhimurium*, *C. difficile*, pathogenic *E. coli* strain, *Campylobacter jejuni*) use fucose or other nutrients (sialic acid, succinate) to grow and spread [95,107,108]. Sometimes the source of nutrition available to both commensals and pathogens changes when the intestinal environment is altered by inflammation or antibiotic treatment [109,110,111]. In such conditions, nutrient type and availability promote the growth of specific pathogens capable of utilizing new nutrient sources. For example, *S. Typhimurium* can use ethanolamine and fructose-asparagine in the inflamed intestine, but the microbiota could not utilize these nutrition sources [112]. The changed microbiota structure and increased specific carbon sources in the mice intestine after antibiotic treatment supported *C. difficile* growth [109]. Pathogenic *E. coli* O157:H7 has developed metabolic pathways for distinct sugar resources, some of which are not available to commensal *E. coli* strains [102]. In the presence of commensal *E. coli*, pathogenic *E. coli* strain may fail to colonize the intestines of mice [101]. Commensal bacteria provide complex sugar resources to some pathogens during competition. For example, *Citrobacter rodentium* requires polysaccharides for successful colonization, whereas monosaccharides alone cannot ensure its survival in competition with other commensals [113]. Therefore, nutrient intake plays a significant role in enteric pathogen pathogenesis. These findings suggest that commensals are well equipped in the healthy intestine to provide colonization resistance to pathogens. The effective colonization of intestinal niches also requires the ability to elude the immune barrier mounted by the mucosal immunity and the antimicrobial metabolites (antimicrobial peptides, secondary bile acids, SCFAs) from microbiota. Similarly, antibiotic treatment induces substantial changes in the intestine microbiota in mice, increasing specific carbon sources that support *C. difficile* growth [109]. These findings indicate that commensals are best equipped to provide colonization resistance to pathogens in the healthy, unperturbed intestines. Several iron-acquisition systems and genes for siderophore production are encoded in the genome of commensal *E. coli* Nissle, which contribute to pathogenicity [114,115]. In addition to iron-acquisition and siderophore production, *E. coli* Nissle developed mechanisms for Curli, Type-1, and F1C fimbriae, which help colonization by increasing adherence and inhibiting other pathogens from colonization [116]. The presence of multiple iron-acquisition systems allows *E. coli* Nissle to compete successfully with *S. Typhimurium* and other enteric pathogens for iron utilization and colonization of inflamed intestines [115]. Alfred Nissle isolated *E. coli* Nissle in 1917 from a soldier stool sample who had not developed diarrhea during the shigellosis outbreak [117]. Currently, probiotic *E. coli* Nissle is used to treat infectious diarrheal diseases and IBD [118]. It may be possible to genetically engineer probiotic commensal strains to compete for nutrients with pathogenic *Enterobacteriaceae* in hostile environments, such as the inflamed intestine [118]. Overall, the beneficial *E. coli* demonstrate how commensals provide colonization resistance to pathogens during the competition for nutrition in the inflamed intestines.

### 6.2. Indirect Mechanisms of Colonization Resistance

Apart from direct colonization resistance, indirect mechanisms of colonization resistance by commensal microbiota are mediated by enhancing the host mucosal barrier and innate and adaptive immune systems to prevent pathogens from colonizing the intestines. This section will explore indirect mechanisms of colonization resistance in detail (Figure 2b).

#### 6.2.1. Epithelial Barrier Enhancement

The intestinal epithelial barrier is a thick layer of epithelial cells. A thin layer of connective tissue between the epithelial and lamina propria promotes healthy communication between the microbiota and immune cells. Immune cells that reside in the intestinal epithelial layer, such as dendritic cells, B cells, T cells, and macrophages, contribute to the integrity of the intestinal epithelial layer [119]. The overlying Peyer’s patches (M cells) and goblet cells secrete mucus in the small intestine to maintain intestinal integrity [120]. The mucus layer that covers and separates the intestinal epithelium from microbiota is the first line of defense. The absence of this protective layer can result in intestinal inflammation, leading to colitis and colorectal cancer [119]. Therefore, a stable microbiota and a healthy mucus layer are essential for preventing bacterial attachment to intestinal epithelial cells. Commensal microbiota resides and metabolizes nutrients in the outer mucus layer of the mouse intestine [121]. In contrast, the thick inner mucus layer of the intestines in humans is firmly attached to the epithelium, preventing direct contact of commensals with epithelial cells and preventing an inflammatory response. This inner mucus layer that separates bacteria from intestinal epithelial cells is thicker in humans than in rodents. The mucosal barrier is compromised in ulcerative colitis patients, and bacteria are directly attached to the inner epithelium [122].

According to recent research, the presence of bacteria affects mucus layer integrity. A Swedish researcher compared germ-free mouse mucus to conventionally-raised mouse mucus and discovered that the mucus level in the intestine of germ-free mice was equivalent to conventionally-raised mice, and the inner intestinal mucus layer in germ-free mice was more permeable to bacteria-sized beads [123]. Other researchers looked at the mucus barrier in the intestines of different wild-type mice and bred mice in germ-free environments. They discovered that genetically identical mice kept in the same environment could have different microbiota and mucus barrier structures. They also discovered that mice with a thicker impenetrable mucus layer had more Erysipelotrichi cells in their intestines, whereas mice with a thin penetrable mucus layer had more *Proteobacteria* and *candidate division TM7* bacteria [124]. Thus, protein synthesis involved in host responses and mucus production can be increased by stimulating intestinal microbiota. These findings show a complex interaction between the intestinal epithelial barrier, microbiota, and host immune system that aids in pathogen tolerance, balancing, or conferring resistance.

#### 6.2.2. Synthesis of Antimicrobial Peptides

Several studies elucidate that commensal microbiota boosts the host immune response to invading pathogens by triggering antimicrobial peptides secretion. Paneth cells and epithelial cells in the small intestine secrete a diverse group of antimicrobial peptides, such as defensin, lysozymes, phospholipases [125], and C-type lectins family islet-derived 3gamma (Reg3γ), Reg3α, and Reg3β [126] to prevent enteric pathogens from colonizing the epithelial cells. These antimicrobial peptides synthesized in the ribosome target both Gram-positive and Gram-negative bacteria [95,127].

#### 6.2.3. Defensins

Based on six conserved cysteine domains, defensins are classified into two subclasses: α-defensins and β-defensins [128]. Paneth cells in the small intestine are the primary producers of α-defensins [125], and colon epithelial cells produce β-defensins [129]. Defensins are essential components of innate immunity, which produce pores in the membranes of targeted bacteria, resulting in loss of membrane integrity and cell death [128]. Compared to α-defensins, β-defensins were active against Gram-positive and Gram-negative enteric pathogens *E. coli*, *S. aureus*, *S. pyogenes*, and *P. aeruginosa* [130].

Evidence shows that intestinal microbiota can stimulate α-defensin production to prevent pathogens colonization within the intestines [48,131]. In a study, live *E. coli* or *S. aureus,* live or dead *S. Typhimurium* derived lipopolysaccharide, lipid A, lipoteichoic acid, and liposomes could stimulate intestinal crypts ability of bacteria or bacteria-derived components to promote α-defensins production from Paneth cells [132]. To investigate whether Paneth cells are in direct contact with the intestinal microbiota to prevent pathogen colonization, Vaishnava and colleagues studied Paneth cells lineage in CR2-MyD88 Tg mouse. They found that Paneth cells sense enteric bacteria directly by activating the MyD88-dependent toll-like receptor (TLR) for α-defensins expression [133]. Lactic acid inhibits α-defensin expression in Caco-2 intestinal epithelial cells in vitro, whereas cecal contents promote α-defensin secretion [134]. The transcript of the α-defensin gene *Defa* was less abundant in the intestinal microbiota of germ-free mice, TLR-deficient mice, and MyD88-deficient mice, but it could be induced by TLR2 or TLR4 stimulation [135]. Miani et al. has reported the relationship between the intestinal microbiome and β-defensin secretion in antibiotic-treated mice and the effect of an aryl hydrocarbon receptor allele on impaired pancreatic β-defensin-14 secretion in non-obese diabetic mice [136]. The findings showed that aryl hydrocarbon receptor ligands, butyrate, and other microbiota-derived compounds were sufficient to induce innate lymphoid cells in the pancreas to secrete interleukin-22 (IL-22), which in turn induced β-defensin-14 secretion by endocrine cells, indicating that dysbiosis and aryl hydrocarbon receptor alleles can influence pancreatic β-defensin-14 secretion in these experimental mice. According to this research study, only live microbiota can promote β-defensin secretion, and specific intestinal microbiota may produce certain metabolites. Aryl hydrocarbon receptor ligands serve as the primary intestinal regulators of β-defensins. However, more in vivo research will be needed to fully understand the mechanisms of β-defensin production in the intestines and how β-defensin prevents pathogen colonization and maintains intestinal homeostasis. Thus, commensal microbiota protects the host by preventing pathogenic bacteria from colonizing the host by activating Paneth cells and colon epithelial cells to produce α-and β-defensin. In addition to defensins, cathelicidins’ antimicrobial peptides with a cathelin domain produced through C-terminal also disrupt the bacterial cell membrane. Cathelicidins, which regulate the microbiota within the colon, are derived from macrophages and colonic epithelial cells in many species, including chickens, fish, humans, mice, and snakes. Researchers have discovered that cathelicidin-WA could improve the intestinal epithelium barrier and protect hosts from enteric pathogens (e.g., *E. coli* O157:H7) colonization [137].

#### 6.2.4. C-Type Lectins Reg3γ, Reg3α, and Reg3β

The C-type lectins (Reg3γ, Reg3α, Reg3β) are key components of innate immunity, which inhibit enteric pathogens growth in the small intestine [138,139]. Paneth cells are the primary producers of Reg3γ in the small intestine [140]. Reg3β is generally co-regulated with Reg3γ in mice [141]. Reg3γ and Reg3β prevent *E. faecalis* [142,143], *Yersinia pseudotuberculosis* [144,145], and *Listeria monocytogenes* from colonizing the small intestine [142]. Additional evidence suggests that Reg3 prevents pathogen colonization in the presence of healthy intestinal microbiota. For example, metronidazole-treated mice exhibited increased Reg3β and Reg3γ expression, increased *E. coli* growth, and decreased *Turicibacteraceae* abundance compared to untreated mice [138]. To further demonstrate how these C-type lectins regulate bacterial composition on the intestinal epithelial surface, Vaishnava et al. reported that Reg3γ deficient mice showed an increased abundance of mucosal-adhered segmented filamentous bacteria without affecting commensals composition in the small intestine [140]. They found no abnormalities in microbial localization within these experimental mice, indicating that Reg3γ can mediate interactions between host tissues and the microbiota. Reg3γ secretion required activation of the MyD88 pathway [146] and recognition of commensal microbiota by TLRs [147]. Earle and colleagues examined the localization of intestinal microbiota and found that removing microbiota-accessible polysaccharides from the diet resulted in distal colonic mucosa thinning, closer microbial proximity to the epithelium, and increased expression level of Reg3β [148]. These findings were consistent with a previous study examining samples from the duodenum, jejunum, ileum, and colon and demonstrated that MyD88 required syncytium endosymbiont-induced colonic epithelial expression of Reg3β and Reg3γ genes. Myd88 deficiency was associated with shaping the microbiota community and increasing the abundance of segmented filamentous bacteria in the small intestine [149].

#### 6.2.5. Interleukins Production can Enhance Pathogens Clearance

Induction of IL-10, IL-17, and IL-22 production by innate immune cells is vital for maintaining mucosal barrier integrity [127,150]. The proinflammatory cytokine IL-22 has been shown to protect the host from a variety of pathogens, including *K. pneumoniae* [151], *C. rodentium* [152], VRE [43], and *Plasmodium chabaudi* [153]. In the presence of antimicrobial peptides, IL-22 can quickly clear the pathogen from the mucosa [154]. Furthermore, evidence showed that specific intestinal microbiota-derived molecules could rapidly induce IL-22 in response to pathogen invasion by activating the host aryl hydrocarbon receptor [155]. For example, *Lactobacillus reuteri* promotes IL-22 expression by type-3 innate lymphoid cells in the intestines [156,157]. Other studies have shown that supplementation with three commensal *Lactobacillus* strains with high tryptophan-metabolizing capability was sufficient to restore IL-22 expression in the intestines [158,159]. These findings show that certain *Lactobacillus* species use tryptophan as an energy source to produce indole-3-aldehyde, which could activate aryl hydrocarbon receptors on intestinal innate lymphoid cells. Other research has shown that *Allobaculum*, *E. coli*, *Clostridium*, and *Bacteroides* can also use tryptophan to produce indole-3-aldehyde and promote IL-22 production. Thus, we can conclude from the above findings that a specific microbiome may help prevent the pathogen from colonizing the intestines by inducing IL-22 expression.

IL-17 is another proinflammatory cytokine that restricts *S. Typhimurium* and *C. albicans* propagation in the intestines by recruiting neutrophils and inducing antimicrobial peptides [127,160]. Studies have shown that commensal microbiota influence both the abundance and activation of IL-17-producing intraepithelial lymphocytes, with enrichment of gamma delta T cells (γδT) being an essential source of IL-17 production [161,162]. Furthermore, a study comparing germ-free mice to specific-pathogen-free mice found that the number of TCRγδ intraepithelial lymphocytes was lower in germ-free mice [127]. Thus, in addition to activating intraepithelial lymphocytes, the intestinal microbiota may also activate TCRγδ intraepithelial lymphocytes in germ-free mice [163]. Moreover, antibiotic treatment of mice revealed a large number of CD62L-γδ T cells (i.e., activated γδ T cells) within the peritonea of specific-pathogen-free mice, whereas germ-free mice showed fewer CD62L-γδ T cells [164]. In conclusion, the intestinal microbiota influences IL-17-producing TCRγδ intraepithelial lymphocytes, which protect the host from pathogen infection and maintain intestinal homeostasis.

The anti-inflammatory cytokine IL-10 regulates the immune response against pathogens and maintains intestinal homeostasis. According to available evidence, macrophages produce IL-10 in the intestines, which plays an essential role in maintaining intestinal homeostasis. Small intestine macrophages have previously been regulated by dietary antigens, whereas the intestinal microbiota regulates colonic macrophages. For example, studies on germ-free mice and specific-pathogen-free mice revealed that colonic lamina propria in germ-free mice produced less IL-10 (by 50%) [165,166]. Morhardt et al. demonstrated that IL-10 is primarily produced by MHCII + CD64 + Ly6C^low^ macrophages in animal models during early injury and is involved in restoring the intestinal epithelial barrier [167]. They found that a lack of IL-10 secretion by macrophages compromised the recovery of the small intestine epithelial barrier. Because the microbiota does not regulate IL-10 production by MHCII + CD64 + Ly6^Clow^ macrophages in the small intestine, microbiota depletion did not affect epithelial recovery. These findings highlight the importance of IL-10-producing macrophages in the recovery of intestinal epithelial injury caused by non-steroidal anti-inflammatory drugs. Hayashi and his colleagues demonstrated that *Clostridium butyricum* induces IL-10 production in macrophage-specific IL-10-deficient mice, which helps to prevent acute colitis. *C. butyricum* treatment, on the other hand, had no effects on IL-10 production from T cells; however, IL-10-producing F4/80 ± CD11b ± CD11c^int^ macrophages accumulated within inflamed mucosa after *C. butyricum* treatment [166]. Subsequently, it has been suggested that dietary amino acids directly regulate the production of IL-10 by small intestine macrophages [168]. A significant decrease of IL-10-producing macrophages in the small intestine was observed in mice fed total parenteral nutrition, but IL-10-producing CD4+ T cells remained unaffected. Similarly, nutrient deprivation reduced IL-10 production by monocyte-derived F4/80+ macrophages but did not affect non-monocyte precursor-derived CD103+ dendritic cells. Unlike colonic macrophages, small intestinal macrophages replenishment and IL-10 production were not regulated by intestinal microbiota. However, the reasons for the disparities in intestinal microbiota observed in different animal model systems remain unknown. Nonetheless, we hypothesized that local damage-associated molecular proteins might regulate immune cells more rapidly and strongly after intestinal damage, resulting in either a failure of intestinal microbiota to adjust or masking any microbiota-based regulatory effect.

This review article thoroughly discussed the effects of antibiotic treatment on pathogen colonization and antibiotic-resistant microorganisms, whereas previous studies did not address this issue in such detail. We also highlighted the importance of the intestinal microbiota and its role in protecting immunocompromised patients from nosocomial infections. In addition, we discussed the clinical short- and long-term consequences of antibiotic treatment and the detailed mechanisms of direct and indirect colonization resistance.

## 7. Conclusions and Future Directions

The intestinal microbiota is a complex and stable microbial community whose close relationship with the host is crucial for maintaining intestinal homeostasis and colonization resistance to pathogens. Disruption of this delicate balance will contribute to disease manifestation. The underlying mechanisms of preventing the pathogen from colonizing the intestines by the intestinal microbiota remain debatable due to the lack of detailed mechanisms and direct evidence. A better understanding of how commensal microbiota interacts with the host is necessary to identify pathogenic and pathophysiological aspects of diseases and to develop a more effective therapeutic agent. FMT therapy, which restores the altered intestinal microbiota to a healthy state, can treat various symptomatic diseases. The benefit of microbiome-based therapy is heavily dependent on the role of dysbiosis in contributing to the disease’s nature. FMT is safe, but it does not guarantee long-term safety because of the risks of transferring antibiotic resistance genes or virulent genes among microbiomes. Although severe systemic side effects from FMT therapy have not been reported, minor GI discomfort such as abdominal pain, vomiting, and nausea are common. As a result, it is strongly encouraged to improve the treatment regimen, administration method, and effective communication with patients. In short, the microbiome research field is still relatively new but rapidly expanding, with several preliminary but promising studies on the modulatory role of the intestinal microbiome in host wellness and diseases. Future research projects in the areas of microbiome-based disease diagnosis, prognosis monitoring, prophylaxis, and treatments have the potential to revolutionize current disease prevention and treatment measures.

## Figures and Tables

**Table 1:** Studies assessing the impact of antibiotic therapy on intestinal microbiota composition.

Study Location	Study Description	Effect on Microbiota Composition	Reference
USA	In a total of 24 healthy volunteers, 8 received amoxicillin (250 mg) three times a day for seven days, and eight controls).	*E. coli* and *Shigella* were the most abundant bacteria, followed by *Bacteroides*, *Clostridium*, *Dialister*, *Coprococcus*, and *Prevotella*, but *Faecalibacterium* species decreased during antibiotic treatment. There was no change in bacterial abundance in the controls.	[169]
USA	Forty-eight households, eight controls were randomly assigned to either amoxicillin (500 mg twice a day), or azithromycin (500 mg on the first day and 250 mg daily).	*Bacteroidaceae*, *Lachnospiraceae* and *Ruminococcaceae* are the most abundant bacterial taxa in the intestines. Amoxicillin treatment significantly decreased *Lachnospiraceae*, *Veillonellaceae*, *Bacteriodales*, and *Porphyromonadaceae* while increasing *Fusobacteriaceae*. *Erysipelotrichaceae*, *Veillonellaceae*, and Clostriales were significantly decreased, whereas *Alcaligenaceae* were increased in response to azithromycin. Those who received amoxicillin therapy for seven days had greater reductions in microbial diversity than those who received it for three days or azithromycin.	[170]
Finland	Fecal samples from 142 children after administering penicillin (amoxicillin with or without clavulanic acid and penicillin V), macrolides (azithromycin and clarithromycin), cephalosporin and sulphonamide-trimethoprim.	*Clostridium*, *Bacteroidetes*, *Dorea*, *Lactobacillales* and *Proteobacteria* increased in response to Macrolide while *Actinobacteria*, *Christensenella* and *Anaerostipes* decreased. Penicillin treatment significantly reduced *Firmicutes*.	[171]
USA	Forty healthy volunteers, before and after seven days of treatment with augmentin (amoxicillin and clavulanic acid) 875 mg twice a day.	*Bacteroides* increased significantly in response to augmentin at 21 days; however, no trend was observed for *Clostridium*, *Bifidobacterium* or *Lactobacillus*.	[172]
Switzerland	In a total of 40 people, ciprofloxacin (500 mg) and nitrofurantoin (100 mg) were given twice a day for five days to ten people.	Ciprofloxacin treatment reduced *Bifidobacterium*, *Alistipes*, *Faecalibacterium*, *Oscillospira*, *Ruminococcus*, and *Dialister*. The abundance of *Bacteroides*, *Blautia*, *Eubacterium* and *Roseburia* increased. Nitrofuratonin treatment increased the number of *Clostridium* species while decreasing *Faecalibacterium*.	[173]
Belgium	Eight UTI patients were treated with nitrofurantoin (100 mg three times a day for 3–15 days), and five control stool samples were analyzed.	Nitrofurantoin treatment had no significant effect on intestinal microbiota except for a slight increase in *Actinobacteria*, which may increase the family *Bifidobacteriaceae*. *Bacteroidetes*, *Firmicutes*, *Proteobacteria*, *Tenericutes* or *Verrucomicrobia* abundance did not change.	[174]
Finland	Ten adults were given doxycycline antibiotics (150 mg per day for ten days), and ten adult controls. In addition, the effect of doxycycline therapy on the *Bifidobacteria* diversity, their susceptibility to tetracycline, and the impact of tetracycline resistance on other bacterial strains were studied.	The diversity of *Bifidobacterium* was significantly higher in the control group than in the antibiotic-treated group. Doxycycline-resistant *Bifidobacteria* species (*B. adolescentis*, *B. ruminantium*, *B. longum*, *B. catenulatum*, *B. pseudocatenulatum*, *B. bifidum* and *B. dentium*) were detected frequently in the doxycycline-treated group. Tetracycline-resistant *Bifidobacterium* isolates were found more frequently in the tetracycline-treated group than in control, indicating that antibiotic treatment increases the population of antibiotic-resistant commensals in the intestines.	[175]
Sweden	In a total of 34 healthy volunteers, 17 were given doxycycline (40 mg once a day for 16 weeks), and 17 given a placebo (controls).	Doxycycline was detectable in stool samples for up to 16 weeks. *Bacteroides*, *Bifidobacterium*, *Clostridium*, *Candida*, *Lactobacillus* and *Enterobacteriaceae* abundance did not change. There has been no new *C. difficile* colonization. Changes in *enterococci* and *E. coli* were observed during the 16-week treatment. There was an increase in doxycycline resistance *Bifidobacterium* species, anaerobic cocci and Gram-positive rods.	[176]
Denmark	Twelve healthy Caucasian males were given broad-spectrum antibiotics (500 mg meropenem, 500 mg vancomycin and 40 mg gentamicin) orally once a day for four days.	Antibiotics treatment increased the abundance of *Enterobacteriaceae* and other pathobionts while decreasing the abundance of *Bifidobacterium* and butyrate-producing species.	[11]
Malaysia	Stool samples from 17 patients treated for seven days with amoxicillin 1000 mg, clarithromycin 500 mg, and pantoprazole 40 mg twice a day.	Even though the general profile of the intestinal microbiome was similar before and after *H. pylori* eradication, some changes in the bacterial communities were noticeable at the phylum and genus levels, with a decrease in *Bacteroidetes* and an increase in *Firmicutes* after *H. pylori* eradication. In addition, there was a significant increase in SCFA-producing bacteria, which could be linked to an increased risk of metabolic disorders.	[24]

## Data Availability

The datasets generated and analyzed during the current study are not publicly available due to the risk of compromising the individual privacy of participants but are available from the corresponding author on reasonable request.
